# Automatic Segmentation of Cervical Cells Based on Star-Convex Polygons in Pap Smear Images

**DOI:** 10.3390/bioengineering10010047

**Published:** 2022-12-30

**Authors:** Yanli Zhao, Chong Fu, Wenchao Zhang, Chen Ye, Zhixiao Wang, Hong-feng Ma

**Affiliations:** 1 School of Computer Science and Engineering, Northeastern University, Shenyang 110819, China; 2 School of Electrical Information Engineering, Ningxia Institute of Science and Technology, Shizuishan 753000, China; 3 Key Laboratory of Intelligent Computing in Medical Image, Ministry of Education, Northeastern University, Shenyang 110819, China; 4 Engineering Research Center of Security Technology of Complex Network System, Ministry of Education, Shenyang 110819, China; 5 Dopamine Group Ltd., Auckland 1542, New Zealand

**Keywords:** computer-aided diagnosis, convolutional neural network, star-convex polygon, segmentation, cervical cytology

## Abstract

Cervical cancer is one of the most common cancers that threaten women’s lives, and its early screening is of great significance for the prevention and treatment of cervical diseases. Pathologically, the accurate segmentation of cervical cells plays a crucial role in the diagnosis of cervical cancer. However, the frequent presence of adherent or overlapping cervical cells in Pap smear images makes separating them individually a difficult task. Currently, there are few studies on the segmentation of adherent cervical cells, and the existing methods commonly suffer from low segmentation accuracy and complex design processes. To address the above problems, we propose a novel star-convex polygon-based convolutional neural network with an encoder-decoder structure, called SPCNet. The model accomplishes the segmentation of adherent cells relying on three steps: automatic feature extraction, star-convex polygon detection, and non-maximal suppression (NMS). Concretely, a new residual-based attentional embedding (RAE) block is suggested for image feature extraction. It fuses the deep features from the attention-based convolutional layers with the shallow features from the original image through the residual connection, enhancing the network’s ability to extract the abundant image features. And then, a polygon-based adaptive NMS (PA-NMS) algorithm is adopted to screen the generated polygon proposals and further achieve the accurate detection of adherent cells, thus allowing the network to completely segment the cell instances in Pap smear images. Finally, the effectiveness of our method is evaluated on three independent datasets. Extensive experimental results demonstrate that the method obtains superior segmentation performance compared to other well-established algorithms.

## 1. Introduction

According to the World Health Organization (WHO), the incidence of cervical cancer in women worldwide is the second highest among female malignancies [1]. There are more than 0.57 million new cases of cervical cancer and about 0.23 million deaths in the world each year, with nearly 80% of them coming from developing countries. Worryingly, the disease has a tendency to develop at a younger age. From a pathological point of view, the incubation period of cervical cancer is generally 8–10 years, during which there are no obvious symptoms [2]. Although it has a high risk, its cure rate is closely related to the disease duration. As long as it is detected early and supplemented with appropriate treatment, the survival rate of cervical cancer patients will be greatly improved. Therefore, early screening and regular examinations of cervical cancer are of great benefit for its diagnosis and subsequent treatment.

The clinical examination of cervical precancerous lesions is mainly based on cervical cytology  [3] (such as Pap test), and it usually requires pathologists to diagnose whether cervical lesions occur through the structural information (such as shape, texture, and staining intensity) of cervical cells. In addition, the canceration of cervical cells is a continuous process, and the visual differences between normal and abnormal cells are small, making it quite tedious and error-prone to screen out abnormal cells from a large number of normal cells. As has been noted in refs. [2,3], traditional manual interpretation and screening methods suffer from high cost, heavy workload, and low reliability. Therefore, to improve the segmentation efficiency of cervical cells, it is necessary and meaningful to explore and develop automated segmentation methods.

**Figure 1:**
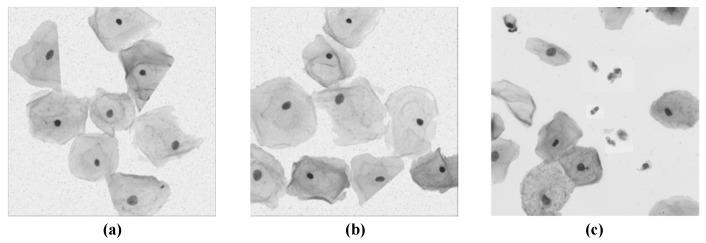
Examples of various challenges. (**a**) The complex structure of cervical cells, including differences in cell shape, color, number, distribution, etc. (**b**) Blurred cell boundaries caused by uneven lighting and staining conditions. (**c**) Blood stains, bacteria, and other impurities remained in the Pap image.

Automatic segmentation of cervical cells contributes to the detection and identification of cervical cancer cells in computer-aided diagnosis (CAD) [4,5,6,7], and it is also the primary task in the entire process of pathological image processing and analysis. However, the task still faces several new challenges, mainly in the following aspects: (1) The complexity of cervical cell structure (e.g., cell color, contour, distribution, and overlap, etc.) makes its segmentation difficult; (2) The production process of smear images, as well as the influence of different staining and lighting conditions, aggravate the blurring of cell boundaries; (3) The presence of impurities such as blood, bacteria, and mucus in Pap images can also affect the segmentation results. Figure 1 provides an example of the challenges facing cervical cell segmentation.

To further identify adherent individuals within complex images, He et al. [8] proposed an instance segmentation algorithm called Mask R-CNN, which first produces region-based proposals by extracting image features, then classifies these proposals and generates the corresponding bounding boxes (bboxs) and masks. Zhang et al. [9] suggested an instance segmentation network for macro-semantic differences, which can model local features through a larger receptive field to generate more discriminative features and effectively reduce the number of network parameters. Although these methods obtain good performance in natural images, they still cannot segment adherent cells well due to the high similarity between cervical cells coupled with the limitation of the standard non-maximum suppression (NMS) [10] with strictly predefined properties. After that, Schmidt et al. [11] presented a polygon-based StarDist method, which is a single-stage instance segmentation approach designed for circular-like objects. It first generates the polygon-based proposals by combining the predicted object probability for each pixel and the corresponding Euclidean distances in different directions. Then, the NMS algorithm is utilized to filter out a final set of polygons that represent object instances. The method differs from the traditional two-stage bbox-based methods. Instead, it employs the star-convex polygons to directly predict and localize targets within images. Additionally, it adopts a simple yet well-performing network structure to segment the adherent targets, which provides a new idea for the segmentation of circular-like objects.

To solve the challenges encountered in the segmentation of adherent cervical cells, considering the excellent performance of the above-mentioned Stardist on circular-like targets, we select the model as the baseline for cervical cell segmentation in this paper. However, it suffers from the following several drawbacks: on the one hand, the down-sampling operation in the feature extraction stage easily leads to the loss of spatial and edge details of images with complex content and backgrounds. On the other hand, the standard NMS for screening proposals in the object detection stage is required to manually set the threshold, which may affect the overall segmentation accuracy. If the threshold is not set properly, it will cause false detection or missed detection. Based on the circular-like characteristics of cervical cells, we thus propose a star-convex polygon-based convolutional network (SPCNet) for the instance segmentation of adherent cervical cells. The model employs the star-convex polygons, generated by jointly predicting the object probability for each pixel belonging to an object and the Euclidean distances of that pixel to the object boundary, to represent and segment cervical cells. Specifically, a newly designed residual-based attentional embedding (RAE) block is introduced into the feature extraction stage to make the network focus on the boundary pixels of adherent cells, thereby improving the segmentation accuracy of cervical cells with the help of the rich contour features obtained. In addition, the polygon-based adaptive NMS (PA-NMS) algorithm is used at the post-processing stage to realize the adaptive setting of the intersection over union (IoU) threshold, so that the polygons that predict adherent targets can be preserved rather than suppressed, thus boosting the final segmentation result.

The contributions of our work can be summarized as follows:

- A star-convex polygon-based SPCNet is proposed for the segmentation of adherent cervical cells. The method utilizes the star-convex polygons to detect objects within Pap smear images and then screens the polygons using a post-processing algorithm to complete the automatic segmentation of cervical cells.
- A residual-based attention embedding block RAE is designed to extract relevant image features. The module provides strong feature extraction and representation capabilities. Moreover, a polygon-based adaptive NMS algorithm is used as the post-processing step of the network to improve the accuracy of cervical cell segmentation.
- The segmentation performance of SPCNet is evaluated on three public datasets. The experimental results demonstrate that our method outperforms other popular algorithms in both segmentation performance and generalization ability.

The rest of this paper is organized as follows. Section 2 introduces the domestic and international technologies and trends related to the research topic. Section 3 describes the overall pipeline of the proposed method, including image pre-processing, network structure, loss function, as well as the adaptive NMS post-processing algorithm. Section 4 explains the detailed implementation of our network and reports the experimental results and performance analyses. Section 5 concludes the paper.

## 2. Related Work

Various segmentation algorithms have been developed to address the issues discussed above over the last few decades. Early ones mainly include threshold method [12], morphological operation [13], K-means [14], level set [15] and gradient vector flow (GVF) [16]. For example, Putzu et al. [17] proposed a cell segmentation method based on color space thresholding. The method can accurately segment cell images with uniform colors but presents a poor segmentation effect on cells with uneven colors. Ruberto et al. [18] utilized a method based on K-nearest neighbors to classify cell images at the pixel level, but the classification result is not satisfactory when the number of samples is unbalanced. Li et al. [19] presented a gradient vector snake model based on prior knowledge, but it offers low segmentation performance for cells with blurred contours due to the small capture range of external forces. Lu et al. [20] used the edge strength function as a shape prior to segmenting adherent cells, but it provides low segmentation accuracy for cell images with complex backgrounds. Therefore, traditional methods cannot solve the segmentation problem of complex cell images, because they mainly rely on the manually extracted low-level features rather than the high-level semantic features representing the cell information in different dimensions. Moreover, the manual-based features have certain limitations and the design process is cumbersome, resulting in a gap between the cell segmentation accuracy and clinical application.

In recent years, deep learning techniques [21,22,23,24,25], which can automatically extract image features and provide strong data representation capabilities, have achieved great success in the field of computer vision [26,27,28,29]. In particular, convolutional neural networks (CNNs) are widely used in medical image processing and analysis. For instance, Long et al. [30] proposed the classic work of applying deep learning to image segmentation tasks - a fully convolutional network (FCN). The network directly employs only convolutional layers to perform the pixel-level classification of images in an end-to-end manner, laying a foundation for the subsequent semantic segmentation. Ronneberger et al. [31] extended FCN and suggested the U-Net model, which adopts the U-shaped encoder-decoder structure to extract and recover image features, and then completes the accurate cell segmentation through multi-level information fusion. Nevertheless, the model is still incapable of segmenting adherent targets under complex image conditions. Chen et al. [32] proposed a deep contour-aware network (DCAN) in the MICCAI Gland and Nucleus Segmentation Challenge. The method makes full use of the multi-layer contextual features and incorporates the multi-task regularization strategy during the training process to enhance the discriminative ability of the intermediate features, and finally accomplishes the adherent object segmentation under an end-to-end multi-task learning framework. However, it still cannot effectively separate the adherent cells with uneven internal grayscale and severe artifacts.

## 3. Methodology

**Figure 2:**
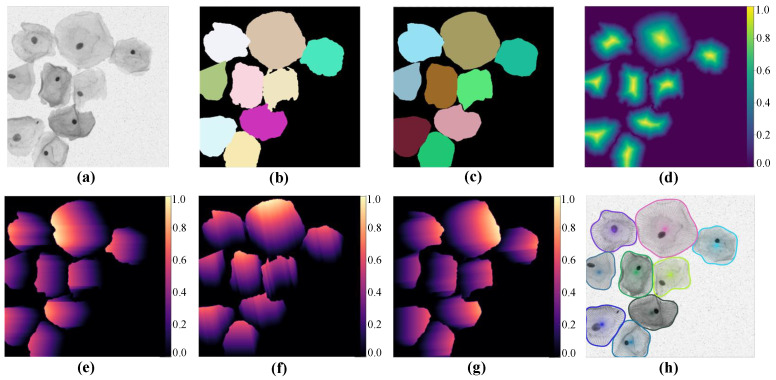
Intermediate results generated by our method. (**a**) An original image and (**b**) its corresponding label. (**c**) The star-convex polygon-based label and (**d**) the object probability map. (**e**–**g**) The normalized Euclidean distance maps for different directions, and (**h**) the final segmentation result.

The presented SPCNet mainly includes three parts in the overall process: image pre-processing for multi-cell labeling, cell segmentation based on star-convex polygons, and NMS post-processing. Figure 2 shows some intermediate results produced by our approach, where Figure 2a,b indicate the sample image and its corresponding original label, respectively. Figure 2c depicts the star-convex polygon-based label, which is annotated by the image pre-processing. Figure 2d denotes the predicted object probability map. Figure 2e–g represents the predicted Euclidean distances in different radial directions, and here only three distance maps are shown, for the sake of simplicity. Figure 2h depicts the final segmented cervical cell instances.

### 3.1. Pre-Processing

**Figure 3:**
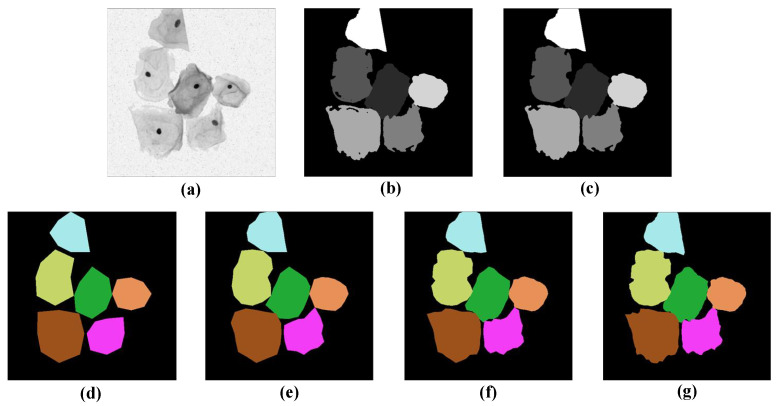
Intermediate results of image pre-processing. (**a**) A sample image, (**b**) the original label with holes, and (**c**) the corresponding filled label. (**d**–**g**) The label images are based on convex polygons with 8, 16, 32, and 64 vertices, respectively.

To apply the star-convex polygon-based method to the segmentation of cervical cells, it is necessary to label the sample images with target probabilities and Euclidean distances in different directions before feeding them into the network. The main pre-processing procedure is as follows: first, a flood-filling algorithm [33] is used to fill the holes existing in the original labels. Next, for the target probability of each sample image, we first classify each pixel as the object or background pixel according to the binary mask, and then define its target probability as the normalized Euclidean distance to the nearest background pixel. After that, for the star-convex polygon distances of each pixel in different radial directions, we first localize the centroid [34] of each cell, and then calculate the Euclidean distances [35] from this point to the boundary of the cell to which it belongs, along the predefined *n* equiangular radial directions. Finally, the corresponding coordinate values for *n* vertices can be computed based on the above *n* radial distances, and then all vertices are sequentially connected to form a star-convex polygon. In this way, the label for each cell within the sample images can be obtained. Figure 3 shows the pre-processing results for a sample image. Figure 3a–c represents the original image, its corresponding label with holes, and the filled label, respectively. Figure 3d–g depicts the labels of star-convex polygons with a different number of vertices (8, 16, 32, and 64 vertices from left to right, respectively). As can be seen that Figure 3f,g can delineate the boundaries of cervical cells more accurately compared to Figure 3d,e. Considering the high computational overhead in Figure 3g, we have to make a trade-off between accuracy and computational cost, and eventually select the star-convex polygons with 32 vertices as labels for the network training.

In addition, to enhance the generalization ability of the model, the labeled sample images discussed earlier need to be expanded by data augmentation techniques [36], mainly including scale transformation, elastic deformation, random rotation, translation, cropping, and flipping. Meanwhile, the grayscale histogram processing [37,38,39,40,41] is also performed on these augmented images to enhance their brightness and contrast. It has an obvious equalizing effect on images with uneven illumination. Then, we resize the training dataset to the same resolution to accelerate the training of the model. More details on the cervical cell dataset can be seen in Section 4.1.

### 3.2. Network Architecture

**Figure 4:**
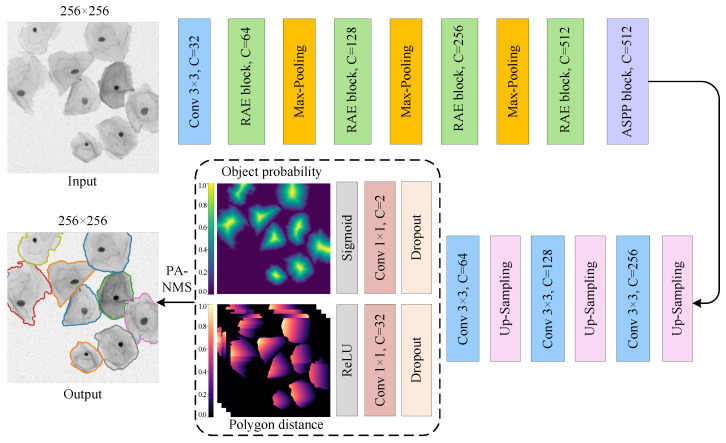
The overall structure of SPCNet, which adopts an encoder-decoder structure and mainly consists of down-sampling and up-sampling paths. Here, the rectangular bars of different colors represent different functional modules, and C denotes the number of channels in the feature map. In the output image, the cell outlines of different colors indicate the segmented cervical cell instances.

Figure 4 depicts the network architecture of the proposed SPCNet, which is a two-branch weight-sharing convolutional network based on the encoder-decoder structure. The model can segment the sample images with an arbitrary resolution, because it is essentially a full CNN composed of different convolutional layers. As shown in Figure 4, our SPCNet takes the single-channel cervical cell images as input, and outputs the segmented images with the same size as the input images. To avoid feature conflicts at the model output caused by only one shared 1×1 convolutional layer before decoupling the object probability and polygon distance prediction tasks, two point-wise convolutions are applied to map the input image features into the single-channel target probability map and the 32-channel normalized Euclidean distance map, respectively. And lastly, the convex polygons formed by the above predictions are screened using the NMS algorithm to obtain the segmented cervical cell instances. Specifically, SPCNet is primarily composed of four types of modules: feature extraction module RAE (green bar), 2×2 maximum pooling down-sampling module (orange bar), bilinear up-sampling module (pink bar), and feature recognition module (blue bar). Among them, the RAE module enhances the feature extraction capability by fusing the residual connection and spatial attention mechanism, and also effectively alleviates the gradient disappearance caused by increasing the network depth.

On the whole, the network structure of SPCNet is essentially symmetrical. In the down-sampling path, the RAE and down-sampling modules are alternately connected, and a total of three down-sampling operations are used to reduce the computational cost while preserving relatively abundant feature information. Besides, considering that the accurate segmentation of cervical cells with various scales usually requires different receptive fields, we connect the ASPP module [42] to the end of the down-sampling path to extract the rich multi-scale features from Pap smear images. Afterward, the output features from the down-sampling path are fed into the up-sampling path, where the up-sampling and feature recognition modules are also alternately connected. After three up-sampling operations, two separate 1×1 convolutional layers are employed to jointly predict the cervical cell contours based on star-convex polygons, and then the predicted results are optimized using the adaptive NMS algorithm. The above strategy ensures that our deep segmentation network SPCNet can achieve satisfactory performance in the segmentation of adherent cervical cells.

#### 3.2.1. RAE Module

**Figure 5:**
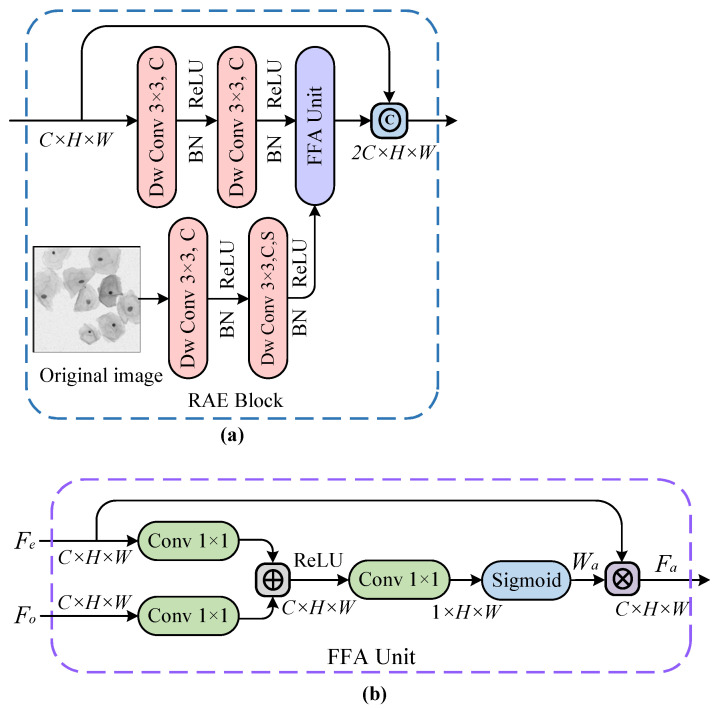
The residual-based attention embedding (RAE) block in (**a**) and the feature fusion attention (FFA) unit in (**b**), where *©*, ⊕ and ⊗ denote the feature map concatenation, pixel-wise addition, and pixel-wise multiplication operations, respectively. And *H*, *W*, *C*, and *S* represent the height, width, number of channels, and convolution stride of the feature map, respectively.

As noted in the previous discussion of the overall network structure, continuous down-sampling operations can easily lead to the loss of position information for the pixels. Therefore, it is necessary to incorporate the shallow features from the original image into deep features from earlier modules during the feature extraction stage, to enhance the model’s feature capturing and representation capabilities. In addition, more focus should be put on the boundaries of cervical cells in direct contact with each other. Given the excellent performance of the attention mechanism [43] in CNNs, we introduce a spatial attention unit into our feature extraction module. The unit progressively suppresses the feature response of irrelevant background regions and focuses on the border pixels of adherent cells, to produce a more discriminative feature representation. Figure 5a shows the RAE module that adopts a parallel structure. Concretely, two successive 3×3 convolutional layers (with ReLU activation functions) are first utilized to extract the shallow features from the original image, and the same operation is used to acquire the deeper features from the earlier modules. After that, the pixel weights of the above feature maps are reconstructed by the feature fusion attention (FFA) unit in Figure 5b to generate space-based attentional feature maps. Finally, a shortcut connection is employed to directly transmit the output of the previous module to the result of the latter module, which not only strengthens the gradient back-propagation during the network training but also improves the network performance.

The FFA unit from the feature extraction module RAE can automatically learn important information about cell structure by capturing the spatial dependencies of features. The unit is performed in the down-sampling path so that only the relevant activation features of coding layers are merged. As depicted in Figure 5b, the FFA unit is used to reconstruct feature maps by combining 1×1 convolution kernels with ReLU and Sigmoid activation functions, where $F_{e}$ denotes the deep feature vector extracted by the encoder and $F_{o}$ indicates the shallow feature vector from the original image. For the input feature vectors $F_{e}$ and $F_{o}$, their non-linear transformations are separately computed using *C* 1×1 convolution kernels followed by the ReLU function, thus enabling $F_{e}$ and $F_{o}$ to be linearly mapped to the $R^{C}$ dimensional space. Next, the features obtained from the previous step are non-linearly transformed into a spatial attention weight map $W_{a}$ through a 1×1 convolution layer followed by the Sigmoid function. Afterward, the weight map $W_{a}$ is multiplied by the corresponding pixels of the input feature map $F_{e}$ to get the final feature map $F_{a}$, which is defined by,

(1) $$\left\{\begin{array}{c} F_{a}=W_{a}\cdot F_{e},\\ \psi_{att}=\varphi^{T}\left(\sigma_{1}\left(W_{e}^{T}F_{e}+W_{o}^{T}F_{o}+b_{o}\right)\right)+b_{\varphi },\\ W_{a}=\sigma_{2}\left(\psi_{att}\left(F_{e},F_{o};\theta_{att}\right)\right), \end{array}\right.$$

where $F_{a}$, $F_{e}$ and $F_{o}$ denote the weighted attention feature vector, the deep feature vector extracted by the encoder and the shallow feature vector from the original image, respectively. And then $\sigma_{1}$ and $\sigma_{2}$ represent ReLU and Sigmoid activation functions, respectively. It is found that the FFA unit described in Equation (1) is mathematically characterized by a set of parameters $\theta_{att}$, which contains the convolution operations $W_{e}$, $W_{o}$ and φ, and bias terms $b_{\varphi }$ and $b_{o}$. Besides, $W_{a}$ indicates the attention coefficient, whose value ranges from 0 to 1, identifies the salient feature regions from encoding layers and suppresses the task-irrelevant feature responses, to retain the activation features associated with cell segmentation. In simple terms, it means that the attention coefficient $W_{a}$ concentrates more on the border portions of adherent cervical cells in low-contrast images.

#### 3.2.2. Loss Function

The design of the loss function is directly related to the model’s final convergence degree. Depending on the task properties of our network, a compound loss function is utilized here as the objective function to jointly train the network model. Specifically, the binary cross entropy (BCE) loss is used for the pixel-wise probability prediction of cervical cells. And the weighted mean absolute error (WMAE) loss is employed for the star-polygon distance prediction of the corresponding pixels. It is weighted by the ground-truth object probability so that predictions for pixels closer to the center of each object are weighted more. To ensure the stability of the network training, these two loss functions are jointly weighted, and the total loss function $L_{total}$ is then defined by,

(2) $$\left\{\begin{array}{c} L_{total}=\alpha L_{BCE}+\beta L_{WMAE},\\ L_{BCE}=-\frac{1}{N}\sum_{i=1}^{N}\sum_{j=1}^{C}y_{i,j}\log x_{i,j},\\ L_{WMAE}=\frac{1}{N}\left[y_{i,j}\sum_{i=1}^{N}\sum_{j=1}^{M}\left|t_{i,j}-p_{i,j}\right|\right], \end{array}\right.$$

where $L_{total}$, $L_{BCE}$ and $L_{WMAE}$ denote the total loss, the binary cross-entropy loss, and the weighted mean absolute error loss, respectively. And then α=0.5 and β=1 represent the weighted coefficients of BCE and WMAE loss functions respectively, $y_{i,j}$ and $x_{i,j}$ indicate the true and predicted results of the object probability for the ith pixel belonging to the *j*th class respectively, and $t_{i,j}$ and $p_{i,j}$ are the true and predicted values of the star-convex distance of the ith pixel, along the *j*th radial direction, to the boundary of the cell to which it belongs, respectively. In addition, *C* is set to 2 in the BCE loss, *M* is set to 32 in the WMAE loss, and *N* denotes the total number of cervical cell pixels within a Pap smear image.

#### 3.2.3. Post-Processing

In view of the fact that the traditional NMS algorithm based on the axis-aligned rectangular bboxes has strong limitations on the detection of convex polygons for circular-like objects. Moreover, the algorithm directly discards the adjacent bboxes with low confidence scores, which easily leads to missed and false detections when similar targets are dense. Therefore, for the star-convex polygons used for the characterization of cervical cells, inspired by Adaptive-NMS [44], we leverage a polygon-based adaptive NMS (PA-NMS) algorithm to perform the detection of convex polygons. Instead of manually setting the IoU threshold, the PA-NMS algorithm defines an output for each object regarding the density of the scene in which it is located, and then takes the output as the IoU threshold to achieve the adaptive threshold setting.

Algorithm 1 shows the pseudocode of the PA-NMS algorithm in Python format. The algorithm flow is as follows: given an image containing many adjacent targets as well as multiple candidate polygon boxes that may overlap with each other for object detection (i.e., each polygon box may represent a certain target), all we need to do is to only retain the best polygon boxes. Suppose there are *n* polygon boxes within an image, each with a score of $s_{i}$ (1 ≤ *i* ≤ *n*) calculated by the classifier, and then we construct four sets *B*, *S*, *D*, and *F*, where *B* is used to store the candidate polygon boxes to be processed, with all *n* boxes initialized; *S* is utilized to store the detection scores of the polygon boxes; *D* is the set that stores densities of the corresponding polygon boxes; And *F* is employed to store the optimal boxes and is initialized to the empty set. Besides, here $N_{t}$ denotes the initial threshold value of the IoU. Concretely, the algorithm is divided into the following four steps: (1) Sort the confidence of all predicted polygons in list *B*, and remove the polygon *M* with the highest score and add it to the target list *F*; (2) Automatically adjust the suppression threshold $N_{M}$ based on the density of the scene where *M* is located; (3) Calculate the polygon-based IoU values by pairing all polygons in *B* with the polygon *M*, and remove the polygons in *B* larger than the threshold $N_{M}$ as well as the corresponding scores in *S*; (4) Repeat the first three steps for all remaining polygons in *B* until the last polygon is left. After the PA-NMS operation, the segmented cervical cell instances are finally obtained, and each polygon represents a cervical cell instance in the Pap smear image.
**Algorithm 1** The polygon-based PA-NMS algorithm**Input:**$B=\left[p_{1},\ldots p_{n}\right],S=\left[s_{1},\ldots s_{n}\right],D=\left[d_{1},\ldots d_{n}\right],N_{t}$ *B* is the list of initial polygon boxes
*S* is the list containing corresponding detection scores
*D* is the list of corresponding detection densities
$N_{t}$ is the initial threshold
F=[]
**while**
B!=[]:
       *m* argmax(*S*)
       M=B[m]
       $N_{M}$ = max $\left(N_{t},d_{M}\right)$
       *F*.append(*M*)
       *B*.remove(*M*)
       **for**
*p*
**in**
*B*:              if polygon_IoU $\left(M,p\right)>=N_{M}$                  B. remove(*p*)                  S. remove(*s*)
**return F,S**


## 4. Experiments

### 4.1. Datasets

A total of three datasets, namely TCC-ISBI, ALL-IDB [45] and EDF-ISBI, are used to test and evaluate the performance of our proposed method. Concretely, TCC is our synthetically generated cervical cell dataset based on the raw samples from ISBI-14 [46]. To validate the feature extraction ability of SPCNet for adherent cervical cells, we randomly synthesize the corresponding cell images according to pre-set parameters such as image size and cell number, so that each image contains 3–9 cells of various numbers and shapes, with only adhesions but no overlaps among them. The dataset includes 3260 single-channel grayscale images of size 256×256 with corresponding ground truths. Additionally, ALL-IDB is a publicly-available blood cell dataset from the University of Milan in Italy, and consists of 108 blood smear images of size 2592×1944 pixels with corresponding labels. Since the limitations of the experimental equipment make it difficult to process high-resolution images, we crop the images from the dataset using the sliding window-based method to make them more compatible with the corresponding equipment conditions, and finally obtain 314 images at the resolution of 512×512 after discarding the sub-images without any targets. Moreover, the EDF-ISBI dataset is created by cropping the original 16 extended depth-of-field (EDF) images of size 1024×1024 pixels from ISBI-15 [46], where each image contains many separated, adherent, and overlapping cervical cells with varying degrees of overlap. Based on the same limitations regarding the experimental equipment, we perform the sliding crop operation on these EDF images to generate sub-images with a resolution of 256×256, and then select 26 sub-images with less overlap as test images.

### 4.2. Evaluation Metrics

To quantitatively assess the segmentation results of our method on adherent cervical cells, we use the pixel-based and object-based indicators [47] as evaluation metrics, with the former containing Dice coefficient (DC), true positive rate (TPp), and false positive rate (FPp), and the latter containing only false negative rate (FN). Besides, considering that an excessive number of false positive objects can affect the accuracy of segmentation results, the average precision (AP) mentioned in ref. [11] is utilized to penalize the false positive detections. The DC and AP indicators, as defined by Equation (3) and Equation (4) respectively, are shown below.

(3) $$DC=2\cdot TP_{p}/\left(2\cdot TP_{p}+FN_{p}+FP_{p}\right)$$

where $TP_{p}$ denotes the correctly identified target pixels, $FN_{p}$ represents the pixels incorrectly identified as background, and $FP_{p}$ indicates the pixels incorrectly segmented as targets. When the value of DC is higher than a predefined threshold τ (usually set to 0.7), the network is considered to obtain good segmentation performance.

(4) $$AP=\frac{TP}{TP+FP+FN}$$

where TP indicates the positive sample that is correctly identified when the IoU of the predicted object and the corresponding label is higher than the threshold τ, FP is the negative sample that is misidentified when the IoU of the predicted object and its label is lower than the threshold τ, and FN denotes the positive sample that is not correctly matched with its label when the IoU value is greater than the threshold τ.

### 4.3. Implementation Details

All experiments are based on the PyTorch deep learning framework and conducted under the Ubuntu 18.04 operating system, with an Intel(R) Core(TM) i7-8700 CPU @ 3.20 GHz with 32 GB RAM, and two NVIDIA GeForce GTX 1080 Ti GPUs. Three datasets described in Section 3.1 are utilized in our experiments: the TCC dataset is used for the model training and testing, and the other two datasets are employed for the model testing only. Note that the TCC dataset needs to be pre-processed according to the method in Section 3.1 before training the network. After that, all sample images are uniformly resized to 256×256 pixels, and then divided into the training and test sets in a ratio of 8:2. In the hyperparameter settings, SGD is selected as the optimizer to train the SPCNet network on the TCC dataset, the momentum and weight decay are set to 0.99 and $1\times 10^{-8}$ respectively, and the initial learning rate and batch size are set to $1\times 10^{-3}$ and 12 respectively, and the number of epochs is 650. Additionally, the threshold for the polygon proposals is set to 0.3, and the IoU thresholds used in the PA-NMS algorithm and the AP indicator are set to 0.4 and 0.7, respectively.

### 4.4. Ablation Study

#### 4.4.1. The Effect of RAE Module on Network Performance

Based on the analysis in Section 3.2, it is clear that down-sampling in CNNs can effectively preserve important image features while avoiding overfitting, but it may lead to the loss of feature information in spatial locations. To this end, a feature extraction module RAE is designed. It allows learning the new information associations of different features through the residual connection and attention mechanism, to enhance the feature information flow among network layers. To test the effectiveness of the module, we use the traditional convolution block (TCB) consisting of two consecutive 3×3 convolutional layers, the residual connection-based TCB block, the attention fusion-based TCB block, and our RAE module as encoders in the down-sampling path of the network, to retrain the network on the TCC dataset, respectively. The experimental results are shown in Table 1.

It is clearly seen from the results in Table 1, that the proposed RAE module combining the residual connection and attention mechanism achieves higher scores on different indicators (e.g., DC, TPp, FPp, FN, and AP), especially reaching 91.86% on the metric of DC, compared with other encoding modules. It indicates that our module can not only obtain the rich shallow information through residual connections, but also focus on and learn the important edge features of cell regions through the spatial attention module, thus demonstrating its strong feature extraction capability, as well as its rationality in terms of structure.

#### 4.4.2. The Effect of ASPP Module on Network Performance

For the presence of cervical cells of various sizes in Pap smear images, we introduce the ASPP module in the down-sampling path of the proposed network to improve the accuracy of cell segmentation. The module mainly consists of multiple parallel atrous convolutional layers with different sampling rates, and it performs well in capturing multi-scale feature information in CNNs. To validate the module’s performance, we retrain the network by using the RAE modules as primary encoders and then connecting the ASPP module to the end of the encoding path of the network. The experimental results are shown in Table 2.

The experimental results show that compared to the network without the ASPP module, the network with the module improves by 0.22% and 0.58% in DC and AP, respectively, as well as a slight increase in other metrics. It demonstrates that the network with the module can extract the abundant multi-scale information of cervical cells using the atrous convolutions with different dilation rates, thus promoting the overall network performance. It also indicates that it is reasonable and meaningful to add the module to our network.

#### 4.4.3. The Effect of PA-NMS Algorithm on Model Performance

Considering that the conventional NMS may discard the better-performing boxes due to the strict culling criteria, and the improper setting of manual thresholds may easily cause missed or false target detections, our polygon-based PA-NMS algorithm is used as the post-processing operation to improve the performance of cervical cell segmentation, based on the RAE and ASPP modules discussed above as the network encoders. The experimental results are shown in Table 3.

The results in Table 3 demonstrate that our PA-NMS algorithm can adaptively adjust the IoU threshold depending on the density of cervical cells, and discard the redundant polygons on the same or adjacent objects according to the threshold. This allows for the accurate detection of all adherent cervical cells in complex scenes, thus increasing the accuracy of the final segmentation. Compared with the NMS algorithm, PA-NMS attains better performance in several metrics, indicating that our post-processing algorithm is effective.

### 4.5. Comparison with Other Popular Models

#### 4.5.1. Evaluation on TCC Dataset

To assess the effectiveness of the constructed SPCNet model, we compare the segmentation performance of the model with other semantic and instance segmentation models on the TCC cervical cell dataset. It is considered that the proposed algorithm is dedicated to extracting the effective features of adherent cervical cells by optimizing the backbone network with a UNet-like structure in the single-stage StarDist, our selected semantic segmentation algorithms mainly include the original U-Net [31], attention-based ATT-UNet [48] and contour aware-based DCAN [32], while the instance segmentation algorithms mainly contain the single-stage StarDist [11] and YOLACT [49], and the two-stage Mask R-CNN [8]. The test results of different models on the TCC dataset are shown in Table 4.

The experimental results indicate that the instance segmentation networks outperform the semantic segmentation networks in the segmentation performance of adherent cervical cells, and it can also be observed that the overall performance of the presented SPCNet is optimal. Specifically, compared with the popular instance segmentation models, our SPCNet has a maximum increase of 4.98%, 3.57%, 0.39%, 6.17%, and 4.86% in DC, TPp, FPp, FN, and AP indicators, respectively. In addition, compared to StarDist, the model improves the above abcd indicators by 3.34%, 2.29%, 0.27%, 2.05%, and 3.41%, respectively, which further proves the effectiveness of our constructed model for the segmentation of adherent cervical cells.

**Figure 6:**
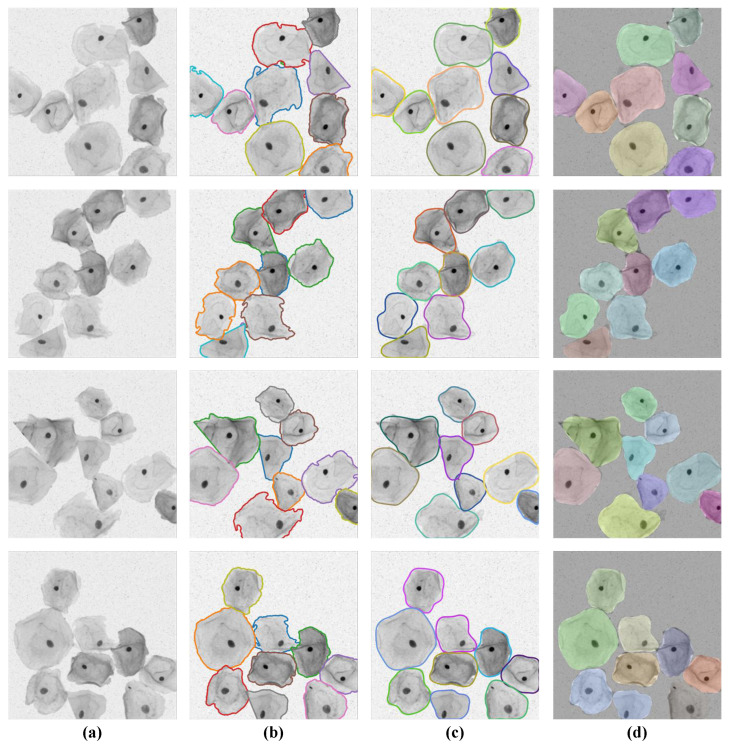
Segmentation results of our SPCNet on the TCC dataset. (**a**) Sample images and (**b**) the corresponding ground truths. (**c**) Predicted results and (**d**) the segmented instances based on random color processing.

The visual segmentation results of the SPCNet model are shown in Figure 6, from which it can be seen that our model can correctly detect the inter-adherent cells in Pap smear images, as well as predict and outline individual cell instances more completely. In particular, for those cervical cell instances with irregular shapes, low contrast of foreground and background, and slight overlap of edge contours, the SPCNet model still sustains excellent performance in accurately separating them from the complex background images.

#### 4.5.2. Evaluation on Other Datasets

To validate the generalization capability of the proposed method, we also assess the segmentation performance of our model on the ALL-IDB and EDF-ISBI datasets. Table 5 shows the performance comparison of our SPCNet and other models on the ALL-IDB dataset. It is clear that our approach presents the best generalization ability compared to other methods. Concretely, compared to the competitive Mask R-CNN, our SPCNet improves by 1.43%, 0.92%, 0.23%, 3.23%, and 1.13% in DC, TPp, FPp, FN, and AP metrics, respectively. Besides, compared with StarDist, the model has an increase of 0.78%, 0.64%, 0.11%, 1.84%, and 0.67% in the above indicators, respectively. It is clear that the suggested method not only offers superior performance but also exhibits strong generalization ability in cervical cell instance segmentation compared to other advanced segmentation methods.

**Figure 7:**
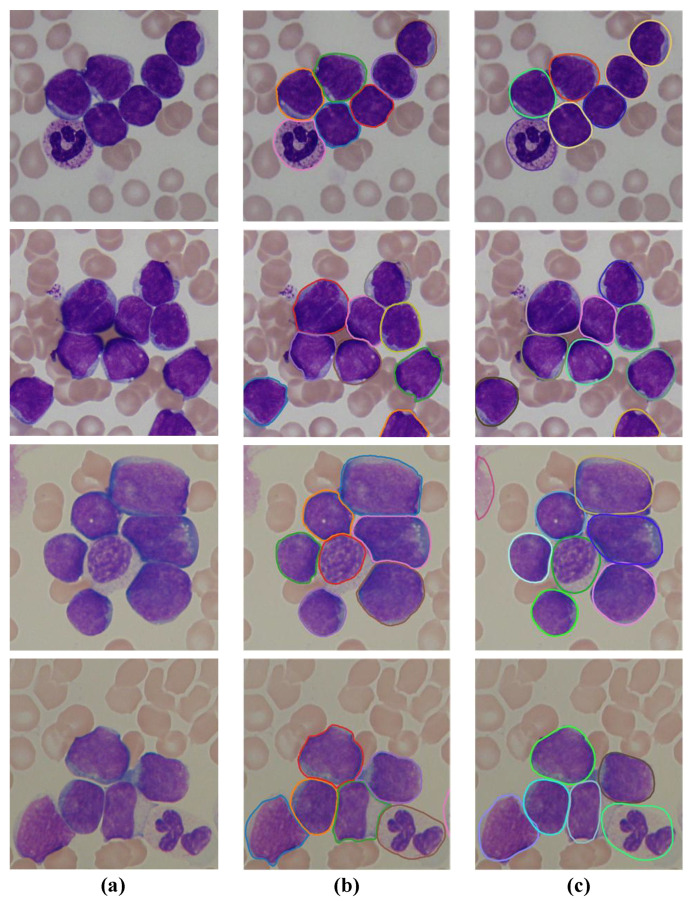
Segmentation results of the SPCNet model on the ALL-IDB dataset. (**a**) Input images, (**b**) the corresponding ground truths, and (**c**) the predicted results.

The visual segmentation results of our algorithm on four sample images from the ALL-IDB dataset are shown in Figure 7. It can be seen that the leukocytes (white blood cells) in blood smear images vary in number, size, and shape, and most of the cells are adherent to each other or slightly overlapping. Based on the obtained segmentation results on the sample images, it can be confirmed that the proposed SPCNet can not only correctly identify the inter-adherent leukocytes, but also accurately depict their edge contours, especially for those cells with relatively blurred edges. It demonstrates that our method can effectively segment the leukocytes in blood smear images, and also proves its strong generalization ability on this dataset.

**Figure 8:**
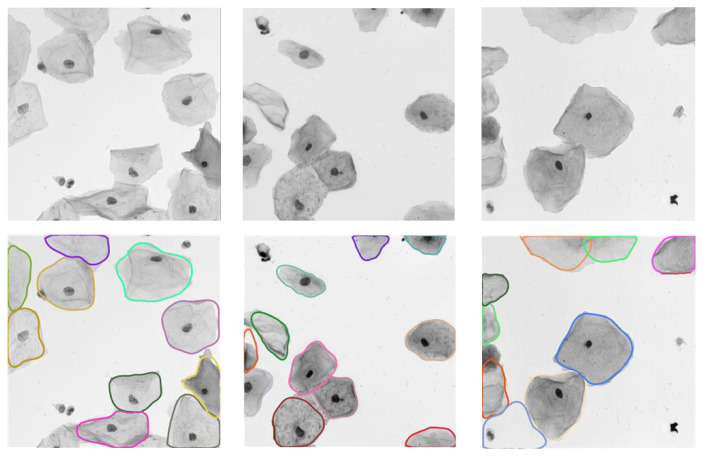
Segmentation results of our model on the EDF-ISBI test set, where the input images and their predictions are shown from top to bottom, respectively.

Figure 8 depicts the qualitative analysis of the proposed method on the EDF-ISBI test set. It is obvious that our algorithm can effectively detect cervical cell instances in real EDF images. Moreover, it can perform the accurate segmentation of cervical cells under complex conditions (e.g., impurities, low-contrast targets, and adherent or overlapping cells). It further validates the strong generalizability ability of our model on other cell image datasets.

## 5. Conclusions

In this paper, we proposed a star-convex polygon-based segmentation method for adherent cervical cells. The method extracts the important feature information of cervical cells, especially the rich contour features, through the attention embedding module based on residual connection, and then utilizes the convex polygon-based PA-NMS algorithm to complete the accurate prediction of cell instances. Experimental results show that our method achieves superior performance on three independent datasets. In future research, considering the importance of accurate segmentation of cervical cells for subsequent classification and diagnosis of cervical cancer cells, there is still a need to optimize our network so that it can be widely used for the segmentation of other medical images.

## Figures and Tables

**Table 1:** The impact of our RAE module on model performance.

Encoders	DC (%)	TPp (%)	FPp (%)	FN (%)	AP (%)
TCB	89.23	84.49	0.46	7.51	86.04
TCB + RC	90.37	84.72	0.41	7.32	86.45
TCB + FFA	91.29	85.68	0.38	7.14	86.72
**RAE (ours)**	91.86	**85.97**	0.31	**6.56**	87.35

**Table 2:** The impact of ASPP module on network performance.

ASPP Module	DC (%)	TPp (%)	FPp (%)	FN (%)	AP (%)
✕	91.86	85.97	0.31	6.56	87.35
*√*	92.08	86.15	0.24	6.15	87.93

**Table 3:** The influence of PA-NMS algorithm on model performance.

Post-Processing	DC (%)	TPp (%)	FPp (%)	FN (%)	AP (%)
NMS	92.08	86.15	0.24	6.15	87.93
PA-NMS (ours)	92.57	86.78	0.19	5.46	89.45

**Table 4:** Performance comparison between our model and the classical segmentation models on the TCC dataset.

Models	DC (%)	TPp (%)	FPp (%)	FN (%)	AP (%)
U-Net	83.34	82.49	0.72	19.32	82.73
ATT-UNet	84.75	82.86	0.66	16.08	83.06
DCAN	85.63	83.05	0.63	13.59	83.71
Mask R-CNN	89.18	84.37	0.37	9.87	85.82
YOLACT	87.59	83.21	0.58	11.63	84.59
StarDist	89.23	84.49	0.46	7.51	86.04
SPCNet (ours)	92.57	86.78	0.19	5.46	89.45

**Table 5:** Performance comparison of our model and the classical segmentation models on the ALL-IDB dataset.

Models	DC (%)	TPp (%)	FPp (%)	FN (%)	AP (%)
U-Net	86.64	84.62	0.68	17.28	83.42
ATT-UNet	87.12	85.07	0.62	16.64	84.27
DCAN	88.34	86.25	0.56	15.88	84.93
Mask R-CNN	92.24	89.76	0.41	9.56	88.96
YOLACT	90.53	87.83	0.47	12.30	86.58
StarDist	92.89	90.04	0.29	8.17	89.42
SPCNet (ours)	93.67	90.68	0.18	6.33	90.09

## Data Availability

The data presented in this study are available on request from the corresponding author.
